# Supplementary material for: MetaRibo-Seq measures translation in microbiomes
Source: Nat Commun. 2020 Jun 29;11:3268. doi: 10.1038/s41467-020-17081-z (PMC7324362; doi:10.1038/s41467-020-17081-z)
Supplement: Supplementary file 10 — Supplementary Data 7 [file 41467_2020_17081_MOESM10_ESM.zip › File2/Confidence_High_Taxonomy/305438_out.krona.html]

Javascript must be enabled to view this page.

members
magnitude
magnitudeUnassigned
count
unassigned
taxon
rank

305438\_out

432

2
superkingdom
432

976
phylum
432

432
200643
class

order
171549
432

family
171552
2

838
genus
1

species
59823

SRS893295\_contig\_number\_7437
1

1
genus
577309


SRS045645\_contig\_number\_4595
species
454154
1

815
family
424

genus
7
424

SRS016954\_contig\_number\_contig-100\_1255.149218SRS019685\_contig\_number\_contig-100\_1384.204031SRS024435\_contig\_number\_contig-100\_11827.158886SRS048870\_contig\_number\_contig-100\_3888.126575SRS076756\_contig\_number\_5322SRS147271\_contig\_number\_contig-100\_1657.210290SRS148784\_contig\_number\_contig-100\_979.138425
816

5

SRS014459\_contig\_number\_2640SRS098571\_contig\_number\_25505SRS098717\_contig\_number\_1170SRS145308\_contig\_number\_1803SRS147088\_contig\_number\_3704
338188
species

14
species
2292956

SRS013940\_contig\_number\_20260SRS014613\_contig\_number\_1579SRS018049\_contig\_number\_1850SRS018427\_contig\_number\_32236SRS019601\_contig\_number\_23164SRS050801\_contig\_number\_3699SRS052078\_contig\_number\_2690SRS064276\_contig\_number\_17734SRS101268\_contig\_number\_2024SRS1041037\_contig\_number\_4845SRS1041039\_contig\_number\_contig-100\_19972.98396SRS1041136\_contig\_number\_14223SRS1041137\_contig\_number\_24701SRS1055076\_contig\_number\_15728


SRS011134\_contig\_number\_44574SRS019601\_contig\_number\_contig-100\_3951.148520SRS019787\_contig\_number\_5223SRS022609\_contig\_number\_31673SRS043001\_contig\_number\_5429SRS046502\_contig\_number\_contig-100\_6266.49591SRS075021\_contig\_number\_contig-100\_1350.58855SRS075878\_contig\_number\_contig-100\_1758.103196SRS077194\_contig\_number\_27901SRS077730\_contig\_number\_18159SRS098571\_contig\_number\_23807SRS1041112\_contig\_number\_6619SRS1041145\_contig\_number\_19863SRS1054928\_contig\_number\_4478SRS893341\_contig\_number\_9770SRS893366\_contig\_number\_21239
2292955
species
16


SRS014923\_contig\_number\_31793SRS024132\_contig\_number\_contig-100\_2560.246669SRS063370\_contig\_number\_2386SRS077294\_contig\_number\_12381SRS1041139\_contig\_number\_4639SRS142599\_contig\_number\_16005SRS144362\_contig\_number\_22773SRS893252\_contig\_number\_12065
457389
species
8

species
2292938

SRS013951\_contig\_number\_38643SRS019161\_contig\_number\_3057
2

1
species
2292941

SRS143181\_contig\_number\_13735

1
species
817

SRS019397\_contig\_number\_83

species
310297

SRS043841\_contig\_number\_4152
1


SRS020394\_contig\_number\_17176SRS045645\_contig\_number\_1322SRS046810\_contig\_number\_contig-100\_0.1SRS054956\_contig\_number\_2718SRS104485\_contig\_number\_172SRS1055034\_contig\_number\_12907SRS142923\_contig\_number\_21808
species
1739517
7

1
585544
species

SRS065176\_contig\_number\_10784

species
28116

SRS011084\_contig\_number\_28781SRS011302\_contig\_number\_20843SRS012849\_contig\_number\_35101SRS012902\_contig\_number\_389SRS013215\_contig\_number\_640SRS013382\_contig\_number\_1843SRS013521\_contig\_number\_4062SRS013638\_contig\_number\_contig-100\_432.128166SRS013687\_contig\_number\_33784SRS014287\_contig\_number\_9552SRS014683\_contig\_number\_18116SRS014736\_contig\_number\_7146SRS014855\_contig\_number\_2259SRS014923\_contig\_number\_42844SRS014979\_contig\_number\_24282SRS015065\_contig\_number\_7448SRS015190\_contig\_number\_9941SRS015431\_contig\_number\_84065SRS015578\_contig\_number\_18604SRS015663\_contig\_number\_contig-100\_1504.193297SRS015782\_contig\_number\_43294SRS015794\_contig\_number\_9750SRS015854\_contig\_number\_3099SRS015890\_contig\_number\_6463SRS016056\_contig\_number\_7381SRS016095\_contig\_number\_30334SRS016132\_contig\_number\_4621SRS016203\_contig\_number\_11416SRS016267\_contig\_number\_3207SRS016381\_contig\_number\_546SRS016840\_contig\_number\_contig-100\_634.34013SRS016989\_contig\_number\_7117SRS017103\_contig\_number\_13389SRS017191\_contig\_number\_3298SRS017247\_contig\_number\_5211SRS017307\_contig\_number\_27981SRS017433\_contig\_number\_13290SRS017622\_contig\_number\_121SRS017701\_contig\_number\_4968SRS017821\_contig\_number\_16331SRS017821\_contig\_number\_19973SRS017859\_contig\_number\_2355SRS018313\_contig\_number\_contig-100\_1113.81247SRS018351\_contig\_number\_2158SRS018623\_contig\_number\_3154SRS018936\_contig\_number\_4156SRS018984\_contig\_number\_2380SRS019030\_contig\_number\_12405SRS019068\_contig\_number\_54445SRS019068\_contig\_number\_88626SRS019161\_contig\_number\_13462SRS019178\_contig\_number\_5091SRS019381\_contig\_number\_568SRS019381\_contig\_number\_2273SRS019496\_contig\_number\_contig-100\_466.105836SRS019693\_contig\_number\_1963SRS019787\_contig\_number\_3204SRS019968\_contig\_number\_14800SRS020271\_contig\_number\_1991SRS021219\_contig\_number\_2704SRS021484\_contig\_number\_26247SRS022071\_contig\_number\_15765SRS022524\_contig\_number\_13964SRS022713\_contig\_number\_1986SRS023346\_contig\_number\_610SRS023715\_contig\_number\_10780SRS023829\_contig\_number\_567SRS023914\_contig\_number\_24120SRS024075\_contig\_number\_2583SRS024075\_contig\_number\_15127SRS024331\_contig\_number\_11637SRS024331\_contig\_number\_28814SRS024549\_contig\_number\_343SRS024549\_contig\_number\_1481SRS024625\_contig\_number\_9511SRS024663\_contig\_number\_744SRS043001\_contig\_number\_1360SRS043411\_contig\_number\_3056SRS043701\_contig\_number\_1795SRS045244\_contig\_number\_477SRS045713\_contig\_number\_5192SRS046068\_contig\_number\_2412SRS046369\_contig\_number\_13206SRS046712\_contig\_number\_1408SRS046717\_contig\_number\_3046SRS048060\_contig\_number\_1293SRS049959\_contig\_number\_21064SRS049995\_contig\_number\_3434SRS050026\_contig\_number\_8601SRS050299\_contig\_number\_12310SRS050422\_contig\_number\_31594SRS050925\_contig\_number\_24562SRS050941\_contig\_number\_22901SRS051031\_contig\_number\_1694SRS052027\_contig\_number\_9815SRS052697\_contig\_number\_6689SRS053398\_contig\_number\_13684SRS053573\_contig\_number\_2181SRS053649\_contig\_number\_583SRS054352\_contig\_number\_22394SRS056273\_contig\_number\_15302SRS056537\_contig\_number\_contig-100\_266.347113SRS057478\_contig\_number\_6142SRS058770\_contig\_number\_13865SRS062427\_contig\_number\_777SRS062701\_contig\_number\_6643SRS063040\_contig\_number\_12039SRS063190\_contig\_number\_2208SRS063813\_contig\_number\_5505SRS063928\_contig\_number\_contig-100\_555.13391SRS064232\_contig\_number\_3582SRS064557\_contig\_number\_473SRS064757\_contig\_number\_2795SRS065504\_contig\_number\_4000SRS074670\_contig\_number\_8859SRS074964\_contig\_number\_7065SRS075078\_contig\_number\_20965SRS075773\_contig\_number\_2218SRS075984\_contig\_number\_13721SRS076976\_contig\_number\_15041SRS077024\_contig\_number\_contig-100\_1727.99647SRS077086\_contig\_number\_contig-100\_789.71507SRS077127\_contig\_number\_2585SRS077194\_contig\_number\_26133SRS077231\_contig\_number\_20514SRS077454\_contig\_number\_2202SRS077502\_contig\_number\_1403SRS077552\_contig\_number\_28790SRS078242\_contig\_number\_29309SRS078665\_contig\_number\_contig-100\_541.77374SRS097889\_contig\_number\_33183SRS097920\_contig\_number\_1495SRS097958\_contig\_number\_contig-100\_382.112769SRS098061\_contig\_number\_8246SRS098655\_contig\_number\_9026SRS101376\_contig\_number\_6483SRS101433\_contig\_number\_14773SRS103987\_contig\_number\_32073SRS103987\_contig\_number\_32530SRS104084\_contig\_number\_4890SRS1041036\_contig\_number\_372SRS1041092\_contig\_number\_12212SRS1041095\_contig\_number\_contig-100\_1263.35360SRS1041118\_contig\_number\_12571SRS1041136\_contig\_number\_contig-100\_3664.149388SRS1041137\_contig\_number\_22922SRS1041142\_contig\_number\_8602SRS1041144\_contig\_number\_1070SRS1041147\_contig\_number\_342SRS104327\_contig\_number\_6822SRS104400\_contig\_number\_48624SRS104693\_contig\_number\_5687SRS104975\_contig\_number\_2799SRS1054928\_contig\_number\_7375SRS1055022\_contig\_number\_11117SRS1055038\_contig\_number\_1277SRS1055056\_contig\_number\_10720SRS1055083\_contig\_number\_12561SRS1055095\_contig\_number\_2172SRS1055099\_contig\_number\_2784SRS140513\_contig\_number\_8968SRS140645\_contig\_number\_567SRS142503\_contig\_number\_54422SRS142542\_contig\_number\_11090SRS143085\_contig\_number\_contig-100\_540.49593SRS143148\_contig\_number\_17390SRS143372\_contig\_number\_1885SRS143417\_contig\_number\_18083SRS143417\_contig\_number\_19679SRS143466\_contig\_number\_18050SRS143598\_contig\_number\_1267SRS143722\_contig\_number\_11067SRS144183\_contig\_number\_36485SRS144297\_contig\_number\_13334SRS144362\_contig\_number\_31040SRS144537\_contig\_number\_contig-100\_3676.218417SRS144603\_contig\_number\_15088SRS145497\_contig\_number\_5970SRS146764\_contig\_number\_33527SRS147039\_contig\_number\_4209SRS147139\_contig\_number\_31293SRS147272\_contig\_number\_640SRS147346\_contig\_number\_3779SRS147377\_contig\_number\_12496SRS147425\_contig\_number\_3790SRS147425\_contig\_number\_6321SRS147614\_contig\_number\_8496SRS148091\_contig\_number\_7240SRS148319\_contig\_number\_118SRS148511\_contig\_number\_564SRS148721\_contig\_number\_16352SRS148817\_contig\_number\_13226SRS148970\_contig\_number\_758SRS149181\_contig\_number\_22163SRS150029\_contig\_number\_3821SRS893170\_contig\_number\_11077SRS893253\_contig\_number\_43SRS893259\_contig\_number\_5846SRS893279\_contig\_number\_9623SRS893285\_contig\_number\_856SRS893292\_contig\_number\_9055SRS893300\_contig\_number\_12531SRS893342\_contig\_number\_10391SRS893358\_contig\_number\_11469SRS893369\_contig\_number\_1733SRS893378\_contig\_number\_3086SRS893382\_contig\_number\_852SRS971276\_contig\_number\_2928
208

1

SRS014412\_contig\_number\_19604
1263050
species

3

SRS013158\_contig\_number\_691SRS143876\_contig\_number\_12604SRS893383\_contig\_number\_19967
species
469590

1
species
162156

SRS013098\_contig\_number\_36487

8

SRS023583\_contig\_number\_7182SRS045826\_contig\_number\_1098SRS057478\_contig\_number\_6935SRS077589\_contig\_number\_4426SRS078419\_contig\_number\_16981SRS1055076\_contig\_number\_175SRS143876\_contig\_number\_2429SRS144506\_contig\_number\_28192
species
47678

1

SRS142980\_contig\_number\_2432
species
2292944


SRS077024\_contig\_number\_11979
species
1262750
1

2302926
species

SRS018575\_contig\_number\_5823SRS056519\_contig\_number\_633SRS075398\_contig\_number\_448SRS076804\_contig\_number\_contig-100\_466.131923SRS1041134\_contig\_number\_919SRS104311\_contig\_number\_43261SRS1055069\_contig\_number\_1015SRS147653\_contig\_number\_6350SRS148159\_contig\_number\_21888SRS148196\_contig\_number\_21076
10

1

SRS893342\_contig\_number\_5881
1263038
species

1

SRS024009\_contig\_number\_1182
species
1796613

457387
species

SRS016267\_contig\_number\_8081SRS016495\_contig\_number\_14617SRS018541\_contig\_number\_10907SRS048164\_contig\_number\_28170SRS053214\_contig\_number\_18500SRS062654\_contig\_number\_2320SRS075341\_contig\_number\_contig-100\_621.141232SRS098644\_contig\_number\_24139SRS143991\_contig\_number\_30524SRS148253\_contig\_number\_contig-100\_3724.49749SRS149879\_contig\_number\_15000
11

371601
species

SRS011405\_contig\_number\_18082SRS012969\_contig\_number\_29667SRS013476\_contig\_number\_27788SRS014235\_contig\_number\_contig-100\_2352.252690SRS014534\_contig\_number\_465SRS015217\_contig\_number\_15247SRS015694\_contig\_number\_contig-100\_1456.139369SRS016132\_contig\_number\_4596SRS016335\_contig\_number\_29626SRS016954\_contig\_number\_contig-100\_1343.149306SRS017247\_contig\_number\_10177SRS017521\_contig\_number\_6378SRS017745\_contig\_number\_1084SRS018049\_contig\_number\_1939SRS018351\_contig\_number\_19968SRS018888\_contig\_number\_9761SRS018984\_contig\_number\_3418SRS019286\_contig\_number\_3609SRS019685\_contig\_number\_contig-100\_505.203152SRS020233\_contig\_number\_27510SRS023526\_contig\_number\_33837SRS023914\_contig\_number\_27708SRS023971\_contig\_number\_559SRS024009\_contig\_number\_4907SRS024625\_contig\_number\_8590SRS042284\_contig\_number\_263SRS043667\_contig\_number\_1858SRS044535\_contig\_number\_22614SRS044535\_contig\_number\_22760SRS047433\_contig\_number\_5695SRS048981\_contig\_number\_1065SRS049402\_contig\_number\_2922SRS049896\_contig\_number\_354SRS050925\_contig\_number\_37113SRS051610\_contig\_number\_2615SRS051882\_contig\_number\_13209SRS052027\_contig\_number\_681SRS052078\_contig\_number\_3076SRS057480\_contig\_number\_contig-100\_1430.76273SRS058070\_contig\_number\_12627SRS058268\_contig\_number\_166SRS058770\_contig\_number\_567SRS063518\_contig\_number\_36489SRS063813\_contig\_number\_contig-100\_353.57584SRS075021\_contig\_number\_4273SRS075716\_contig\_number\_2511SRS076804\_contig\_number\_contig-100\_959.132416SRS077849\_contig\_number\_20831SRS097889\_contig\_number\_33360SRS098073\_contig\_number\_13374SRS101268\_contig\_number\_1973SRS1041116\_contig\_number\_2954SRS1041164\_contig\_number\_1002SRS104912\_contig\_number\_12975SRS1055049\_contig\_number\_15769SRS1055083\_contig\_number\_12701SRS142890\_contig\_number\_31744SRS143780\_contig\_number\_4331SRS146813\_contig\_number\_3817SRS147377\_contig\_number\_12697SRS149325\_contig\_number\_677SRS893285\_contig\_number\_1961SRS893380\_contig\_number\_267
63


SRS045739\_contig\_number\_264SRS047014\_contig\_number\_15888SRS098644\_contig\_number\_11783SRS1041130\_contig\_number\_5
species
46506
4

2302925
species

SRS063489\_contig\_number\_6390
1


SRS011405\_contig\_number\_3760SRS045004\_contig\_number\_3566SRS049446\_contig\_number\_3051SRS057717\_contig\_number\_1512SRS147977\_contig\_number\_942SRS149075\_contig\_number\_1308SRS893256\_contig\_number\_412
818
species
7

1
246787
species

SRS065397\_contig\_number\_13511

species
556259

SRS011239\_contig\_number\_23519SRS013687\_contig\_number\_14179SRS013800\_contig\_number\_852SRS014235\_contig\_number\_44892SRS016056\_contig\_number\_7362SRS016753\_contig\_number\_4283SRS017307\_contig\_number\_27974SRS020233\_contig\_number\_58778SRS020508\_contig\_number\_795SRS020869\_contig\_number\_23392SRS021153\_contig\_number\_1448SRS022137\_contig\_number\_21411SRS022713\_contig\_number\_4819SRS024492\_contig\_number\_19179SRS045195\_contig\_number\_6868SRS045713\_contig\_number\_9972SRS048981\_contig\_number\_6843SRS049773\_contig\_number\_16738SRS050752\_contig\_number\_30732SRS052576\_contig\_number\_contig-100\_313.15822SRS054905\_contig\_number\_11895SRS055982\_contig\_number\_1199SRS1041090\_contig\_number\_1793SRS1041138\_contig\_number\_2949SRS104311\_contig\_number\_18352SRS1055067\_contig\_number\_15535SRS142781\_contig\_number\_5362SRS143523\_contig\_number\_1310SRS143895\_contig\_number\_15508SRS146812\_contig\_number\_30635SRS146813\_contig\_number\_29480SRS147022\_contig\_number\_1520SRS147919\_contig\_number\_5299SRS148874\_contig\_number\_16761SRS892882\_contig\_number\_15075SRS893341\_contig\_number\_15342SRS893373\_contig\_number\_27607
37

2
species
821

SRS064973\_contig\_number\_9SRS893288\_contig\_number\_1667

2005525
family
4


SRS024388\_contig\_number\_8773
375288
4
genus
1

1

SRS064557\_contig\_number\_64
species
46503

2293113
species

SRS055966\_contig\_number\_1610SRS063127\_contig\_number\_875
2

2
1853231
family

2
283168
genus

2

SRS047014\_contig\_number\_30527SRS140492\_contig\_number\_15165
28118
species
